# Rolling Bearing Fault Diagnosis Based on CKEPM Encoding and Tri-CrossViT

**DOI:** 10.3390/s26206639

**Published:** 2026-10-20

**Authors:** Xingyue Cui, Yueqian Bian, Xiaoguang Wu, Tianwen Guo

**Affiliations:** College of Computer and Information Engineering, Nanjing Tech University, Nanjing 211816, China

**Keywords:** rolling bearing fault, Complete Ensemble Empirical Mode Decomposition with Adaptive Noise, Markov Transition Field, triple-scale

## Abstract

Rolling bearings are indispensable components in mechanical equipment, and their condition directly affects the operational reliability and maintenance costs. To address the requirements of high-precision and robust bearing fault diagnosis, this paper proposes a dynamic-mapping method named CKEPM, based on Complete Ensemble Empirical Mode Decomposition (CEEMDAN), kurtosis-, energy-, and Pearson-correlation-based intrinsic mode function (IMF) selection, followed by Markov Transition Field (MTF) encoding. Furthermore, a triple-scale, cross-branch network Tri-CrossViT is constructed to hierarchically model fault-related features at different resolutions and enable cross-scale information interaction for bearing fault classification. Specifically, vibration signals are first decomposed using CEEMDAN and the fault-sensitive intrinsic mode functions are selected based on kurtosis, energy, and Pearson correlation criteria. Then, these components are encoded by the Markov Transition Field to form RGB time–frequency images. Subsequently, the proposed Tri-CrossViT processes the generated images through three parallel branches with different patch resolutions to capture fault-related information at triple scales, including localized impulsive transients, modulation-related envelope structures, and global spectral energy distributions, and a Cross-branch attention is introduced to fuse complementary fault information across scales, enabling joint learning of local impulses and global dynamic characteristics. The Tri-CrossViT achieves diagnostic accuracies of 98.87% and 99.51% on the Case Western Reserve University (CWRU) and Paderborn University (PU) datasets, respectively, realizing outstanding classification performance. The experimental results demonstrate the effectiveness of the integrated CKEPM-Tri-CrossViT framework for rolling bearing fault diagnosis under the evaluated benchmark conditions.

## 1. Introduction

Rolling bearings are critical components in rotating machinery and are widely used in industrial systems such as electric motors, gearboxes, wind turbines, and ship propulsion. Their operating condition directly affects equipment reliability and operating cost. When defects occur on raceways, rolling elements, and cages, vibration and noise usually increase abnormally, which may lead to reduced efficiency or even safety incidents. Therefore, developing efficient and reliable bearing fault diagnosis methods is crucial for enhancing the reliability and safety of equipment operation [1,2].

Early diagnostic studies largely relied on handcrafted time-domain and frequency-domain features (e.g., statistical indicators, spectral/envelope analysis) coupled with shallow classifiers. However, when working conditions vary (load/speed) or interference becomes significant, feature engineering and shallow learners often degrade due to insufficient representation of nonlinear coupling and domain shift. Recent studies have further indicated that variations in operating conditions and distribution differences between training and test data remain important challenges for bearing fault diagnosis [2,3,4].

With the rapid development of deep learning, intelligent fault diagnosis methods have gradually shifted from handcrafted feature extraction toward automatic representation learning. Recent studies have explored advanced deep learning frameworks for industrial fault monitoring and condition recognition, demonstrating the capability of neural networks to extract discriminative information from complex measurement data [5,6]. Nevertheless, recent reviews have indicated that the diagnostic performance of deep learning models can still be affected by complex working conditions and limitations in feature representation [1]. These developments further highlight the potential of deep learning-based approaches for improving machinery condition monitoring. However, effectively capturing fault-related multi-scale characteristics and modeling complex dependencies in vibration signals remain challenging problems.

To enhance the discriminability of fault information, many studies convert one-dimensional (1D) vibration signals into two-dimensional (2D) time–frequency (or time–frequency-like) maps and then perform image-based learning with CNNs. For example, Zhang et al. used time–frequency images as CNN inputs and improved classification performance via an enhanced CNN design tailored to such representations [7]. Chen et al. combined cyclic spectral coherence (CSCoh) maps with CNNs to obtain stronger discriminative representations under varying operating conditions [8]. Yuan et al. used CWT-based time–frequency images and integrated CNN features with an SVM decision stage to improve diagnosis when training samples are limited [9]. More recently, CEEMDAN-based signal decomposition has been combined with CNN-based diagnosis to reduce noise interference and enhance fault feature extraction [10]. MTF-based approaches have also been employed to transform one-dimensional vibration signals into structured two-dimensional representations while retaining temporal transition information [11]. Furthermore, Ding et al. combined MTF encoding with a multi-scale Runge–Kutta residual attention network to extract multi-scale fault information under variable working conditions [12]. Although these approaches effectively exploit local spatial learning, they remain constrained by the inherent locality of convolution and may be sensitive to preprocessing techniques and parameter choices in complex regimes. Moreover, the effectiveness of image-based diagnosis depends not only on the subsequent classifier but also on whether the generated representations retain sufficiently discriminative fault information.

Recently, Transformer-based architectures have been explored for bearing diagnosis because the attention mechanism enables global interaction within images (or time–frequency bins), which is beneficial when fault evidence is distributed across time–frequency bands. Ding et al. proposed a time–frequency Transformer to strengthen global modeling capability for rolling bearing diagnosis [13]. Dai et al. combined signal decomposition and image encoding with a Swin Transformer-based transfer learning strategy to improve performance across varying operating conditions [14]. Furthermore, Wang et al. designed a multi-scale Transformer-style fusion framework to integrate time–frequency information for rolling bearing fault diagnosis [15]. More recently, a deformable convolution-based Vision Transformer (DEViT) was developed to enhance local feature extraction while retaining the global modeling capability of Transformer architectures [16]. However, existing Transformer-based diagnostic methods still face challenges in effectively coordinating local fault details with global dependencies and exploiting complementary fault information across multiple feature scales.

Thus, to reconcile local-detail extraction with global contextual reasoning, multi-scale and multi-branch Vision Transformer architectures have been proposed, where parallel token streams at different patch scales exchange information through cross-attention. For instance, CrossViT explicitly couples small- and large-patch branches using cross-attention for complementary fusion of fine-grained and coarse representations [17]. Swin Transformer introduces hierarchical representations with window-based self-attention to scale global modeling while preserving locality [18]. Pyramid Vision Transformer (PVT) further supports multi-scale token hierarchies to strengthen representation across resolutions [19]. In the field of bearing fault diagnosis, Wang et al. further developed a multi-scale TransFusion network that integrates time–frequency information and exploits Transformer-based global dependency modeling under multiple operating conditions [15].

Overall, the reviewed studies reveal two complementary research directions in intelligent bearing fault diagnosis. One direction focuses on improving the representation of non-stationary vibration signals through signal decomposition and two-dimensional encoding, while the other focuses on enhancing feature learning through attention-based and multi-scale network architectures. Despite the progress achieved in both directions, the selection and integration of complementary fault-sensitive components during feature construction and the coordinated modeling of fault characteristics at different scales still warrant further investigation. In particular, integrating fault-sensitive signal representation with cross-scale feature interaction provides a meaningful direction for simultaneously improving feature quality and multi-scale representation capability.

Motivated by the above research gaps and the multi-scale nature of bearing fault signatures, this paper proposes a CKEPM-based feature extraction scheme together with a triple-scale cross-branch Vision Transformer (Tri-CrossViT) for bearing fault diagnosis. Specifically, the raw vibration signals are first decomposed into IMFs using CEEMDAN to alleviate mode mixing and improve decomposition stability. Then, the most fault-informative IMFs are selected according to a joint criterion combining kurtosis, signal energy, and Pearson correlation with the original signal, ensuring the retention of impulsive, energetic, and structurally correlated components while suppressing the redundant or noise-dominated modes. Following this, the selected IMFs are encoded into 2D representations using MTF, which preserves temporal transition dynamics and transforms the 1D signal into a structured image domain. Finally, by mapping the MTF representations of the selected IMFs to the R, G, and B channels, an RGB time–frequency dynamic-structure image that explicitly highlights complementary fault characteristics across channels is generated by CKEPM. Building on this representation, Tri-CrossViT constructs parallel token branches at small scale (to emphasize high-frequency impulsive events), medium scale (to capture modulation-related structures and local textures), and large scale (to model global frequency-energy distributions), and performs cross-branch feature interaction through cross-attention. Comparative experiments are conducted on the CWRU and PU bearing fault datasets to verify the effectiveness of the proposed method.

The main contributions of this work are summarized as follows:

(1) A fault-sensitive signal representation method, termed CKEPM, is developed by integrating CEEMDAN, multi-criterion IMF selection, and MTF encoding. Specifically, kurtosis, energy, and Pearson correlation are jointly employed to select complementary fault-sensitive IMFs, which are subsequently encoded by MTF and mapped to different RGB channels. This strategy enables complementary vibration characteristics to be preserved within a unified two-dimensional representation.

(2) A triple-scale cross-branch Vision Transformer, termed Tri-CrossViT, is proposed for multi-scale bearing fault feature learning. Three parallel branches with different patch resolutions are employed to capture fault characteristics at different spatial scales, while intermediate cross-scale attention enables information exchange among branches, thereby integrating complementary local and global fault information.

(3) An integrated CKEPM–Tri-CrossViT diagnostic framework is established and systematically evaluated on the CWRU and PU bearing datasets. Comparative experiments with representative deep learning models, feature-encoding methods, and ablation studies are conducted to evaluate the effectiveness of the proposed signal representation and cross-scale interaction strategy.

The remainder of this paper is organized as follows. Section 2 introduces the proposed CKEPM feature extraction method, including signal segmentation, CEEMDAN decomposition, multi-criterion IMF selection, MTF encoding, and RGB feature construction. Section 3 presents the proposed Tri-CrossViT architecture and describes its major components, including the TriScalePatchEmbed, Branch Transformer, Tri-CrossAttention, and Fusion Head modules. Section 4 describes the experimental setup and presents the comparative experiments and ablation studies on the CWRU and PU bearing datasets. Finally, Section 5 summarizes the main findings, discusses the limitations of the present study, and outlines directions for future work.

## 2. Feature Extraction Based on CKEPM

**Figure 1:**
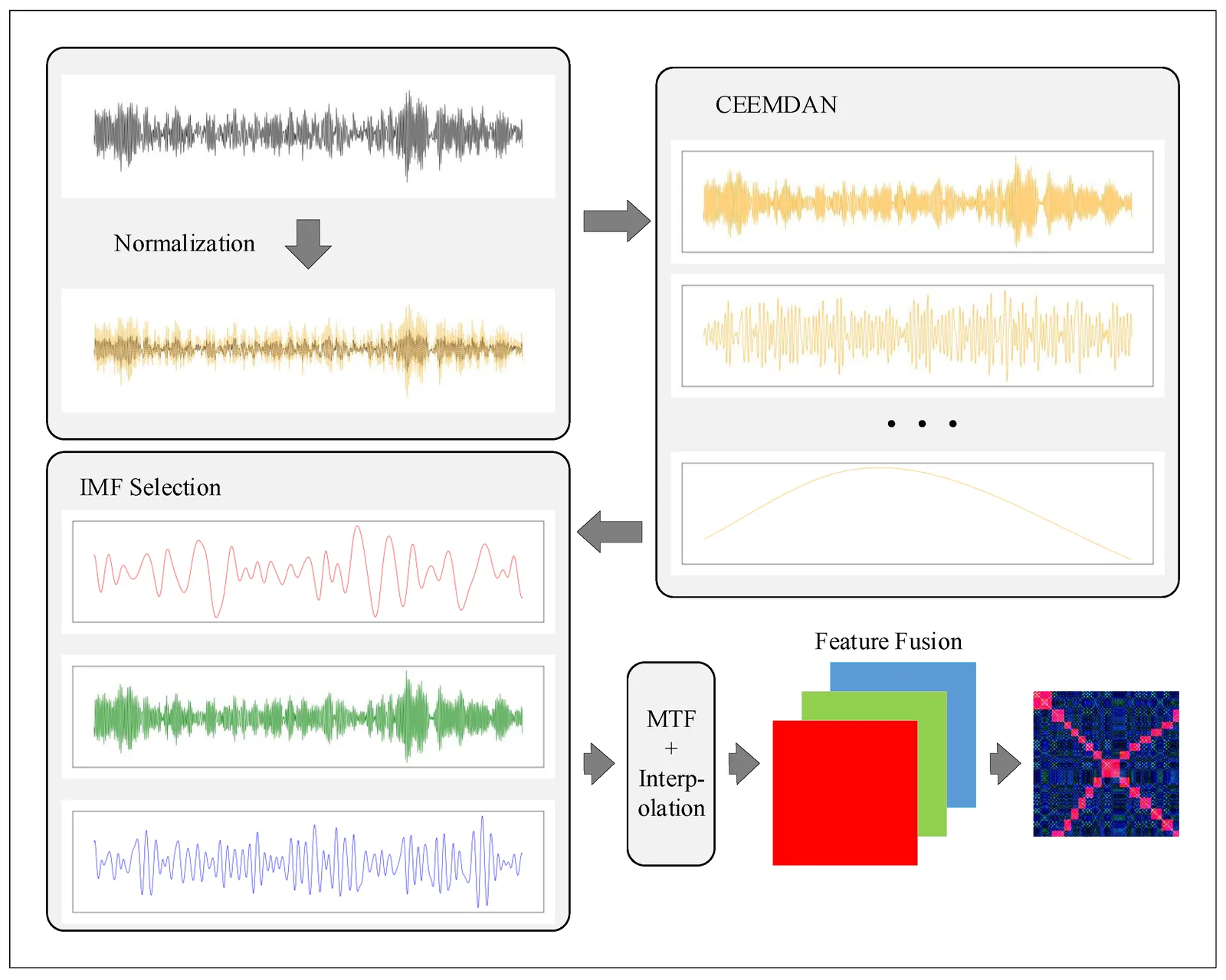
Overall workflow of the proposed CKEPM feature extraction method. The raw vibration signal is segmented and normalized before CEEMDAN decomposition. The resulting IMFs are evaluated using kurtosis, energy, and Pearson correlation, and three non-repetitive fault-sensitive IMFs are selected. These IMFs are subsequently transformed using MTF, resized to 224 × 224, and assigned to the R, G, and B channels, respectively, to construct the final CKEPM representation.

The proposed CKEPM method consists of three main stages, as illustrated in Figure 1. First, each segmented and normalized vibration signal is decomposed into a set of IMFs using CEEMDAN. Second, the resulting IMFs are evaluated using kurtosis, energy, and Pearson correlation, and three non-repetitive fault-sensitive IMFs are selected according to the predefined priority order of kurtosis > energy > Pearson correlation. Third, the selected IMFs are individually encoded using MTF and resized to 224 × 224. The MTF representations corresponding to kurtosis-, energy-, and Pearson-correlation-based IMF selection are assigned to the R, G, and B channels, respectively, to construct the final CKEPM RGB representation. Figure 1 provides an overview of the complete data flow from the original vibration signal to the CKEPM representation used as the input of Tri-CrossViT.

### 2.1. CEEMDAN

Complete Ensemble Empirical Mode Decomposition with Adaptive Noise (CEEMDAN) [20] reduces residual noise and enhances decomposition stability by integrating an adaptive noise-injection strategy with an iterative decomposition-and-averaging mechanism. The core advantages include: The added noise is reduced incrementally, resulting in smaller residual noise; the noise is dynamically adjusted during decomposition to avoid spurious modes; each IMF is immediately averaged after generation to ensure component alignment.

Given the original signal y(t), CEEMDAN processing is as follows:

(1) Add *N* times Gaussian white noise to the original signal y(t) to construct *N* preprocessed sequences $y_{n}\left(t\right)$:

(1) $$y_{n}\left(t\right)=y\left(t\right)+\varepsilon_{0}\delta_{n}\left(t\right)$$

where n=1,2,…,N, and $\delta_{n}\left(t\right)$ is the *n*-th realization of Gaussian white noise.

(2) Apply EMD to all preprocessed $y_{n}\left(t\right)$ to obtain the first IMF $c_{1}^{n}\left(t\right)$. Take the ensemble average of $c_{1}^{n}\left(t\right)$ for all $y_{n}\left(t\right)$ as the first IMF of CEEMDAN, and compute the first residual sequence $r_{1}\left(t\right)$:

(2) $$c_{1}\left(t\right)=\frac{1}{N}\sum_{n=1}^{N}E_{1}\left(y_{n}\left(t\right)\right)$$

(3) $$r_{1}\left(t\right)=y\left(t\right)-c_{1}\left(t\right)$$

(3) Similarly, Gaussian white noise, processed through EMD to extract its first IMF, is added to the residual $r_{1}\left(t\right)$ to form *N* new sequences. Perform EMD on these sequences and take their mean to obtain the second IMF of CEEMDAN $c_{2}\left(t\right)$, and then compute the second residual $r_{2}\left(t\right)$ as

(4) $$c_{2}\left(t\right)=\frac{1}{N}\sum_{n=1}^{N}E_{1}\left(r_{1}\left(t\right)+\varepsilon_{1}E_{1}\left(\delta_{n}\left(t\right)\right)\right)$$

(5) $$r_{2}\left(t\right)=r_{1}\left(t\right)-c_{2}\left(t\right).$$

By induction, the *m*-th IMF and residual can be obtained as

(6) $$c_{m}\left(t\right)=\frac{1}{N}\sum_{n=1}^{N}E_{1}\left(r_{m-1}\left(t\right)+\varepsilon_{m-1}E_{m-1}\left(\delta_{n}\left(t\right)\right)\right)$$

(7) $$r_{m}\left(t\right)=r_{m-1}\left(t\right)-c_{m}\left(t\right)$$

where $\varepsilon_{p}$ denotes the noise scaling coefficient at the *p*-th decomposition stage, and $E_{p}$ denotes the *p*-th IMF extracted by applying EMD. In particular, $\varepsilon_{0}$ corresponds to the noise scaling coefficient at the initial stage (*p* = 0), used for constructing the noise-added signal.

(4) Repeat the above steps until the residual becomes a monotonic sequence. The original signal y(t) after CEEMDAN can then be expressed as

(8) $$y\left(t\right)=r_{M}\left(t\right)+\sum_{m=1}^{M}c_{m}\left(t\right)$$

where *M* denotes the total number of extracted IMFs.

### 2.2. IMF Selection

The statistical measures used for IMF selection are kurtosis, energy, and the pearson correlation coefficient. For statistical computation, the IMF $c_{m}\left(t\right)$ is sampled at discrete time instants $t_{k}$, forming a discrete sequence $c_{m}\left(t_{k}\right),k=1,\ldots ,L$; the original signal y(t) is also sampled at discrete time instants $t_{k}$, forming a discrete sequence $y(t_{k}),k=1,\ldots ,L$, where *L* denotes the number of samples.

Kurtosis measures the peakness of the signal, which is highly sensitive to fault-impact signals, and therefore can be used to detect and diagnose early surface-damage-type faults. For an IMF $c_{m}\left(t_{k}\right)$, the kurtosis is defined as

(9) $$K_{m}=\frac{\frac{1}{L}\sum_{k=1}^{L}{\left(c_{m}\left(t_{k}\right)-\bar{c_{m}}\right)}^{4}}{{\left(\frac{1}{L}\sum_{k=1}^{L}{\left(c_{m}\left(t_{k}\right)-\bar{c_{m}}\right)}^{2}\right)}^{2}}$$

where $\bar{c_{m}}=\frac{1}{L}\sum_{k=1}^{L}c_{m}\left(t_{k}\right)$ is the mean value of the *m*-th IMF. Usually, A larger kurtosis indicates that the IMF contains more pronounced impulsive features.

Signal energy measures the signal strength or instantaneous activity. For an IMF $c_{m}\left(t_{k}\right)$, the energy is defined as

(10) $$E=\sum_{k=1}^{L}c_{m}{\left(t_{k}\right)}^{2}.$$

Bearing faults often produce transient impacts that significantly increase the energy of certain IMFs.

Besides, to evaluate how well each IMF preserves fault-related features, the Pearson correlation coefficient is used to measure the correlation between each IMF and the original signal. It is defined as

(11) $$\text{Coefficient}=\frac{\sum_{k=1}^{L}\left(c_{m}\left(t_{k}\right)-\bar{c_{m}}\right)\left(y\left(t_{k}\right)-\bar{y}\right)}{\sqrt{\sum_{k=1}^{L}{\left(c_{m}\left(t_{k}\right)-\bar{c_{m}}\right)}^{2}}\sqrt{\sum_{k=1}^{L}{\left(y\left(t_{k}\right)-\bar{y}\right)}^{2}}}$$

where $\bar{y}$ is the mean value of the original signal segment.

The IMF selection rules are as follows: To avoid information redundancy when multiple channel sources come from the same IMF, the priority order of the three selection criteria is: kurtosis > energy > Pearson correlation coefficient. If a lower-priority criterion selects the same IMF as a higher-priority criterion, the lower-priority criterion should select the next-best IMF.

### 2.3. MTF-Based IMF Fusion

The selected IMFs provide a compact yet informative representation of fault-related oscillatory components. To further characterize their temporal transition dynamics, the Markov Transition Field (MTF) [21] is employed to transform each selected one-dimensional IMF into a two-dimensional representation. MTF encodes transition probabilities between discretized signal states while preserving their temporal positions.

For a selected IMF $c_{m}\left(t_{k}\right)$, the signal amplitude sequence is first discretized into *Q* quantile-based states 1,2,…,Q. Then, each sample $c_{m}\left(t_{k}\right)$ is assigned to a corresponding quantile state based on its amplitude. This discretization transforms the continuous-valued discrete-time signal into a symbolic state sequence $s_{k}\in \left\{1,2,\ldots ,Q\right\}$, k=1,…,L.

Based on the state sequence, a first-order Markov transition matrix $P^{\left(m\right)}\in R^{Q\times Q}$ is constructed, where each element is defined as

(12) $$P_{ij}^{\left(m\right)}=\mathrm{Pr}\left(s_{k+1}=j|s_{k}=i\right)$$

where $P_{ij}^{\left(m\right)}$ denotes the probability of transitioning from state *i* to state *j*. The transition probabilities are estimated by counting state transitions along the time axis and normalizing each row of the matrix.

To preserve the temporal ordering of transitions, the Markov Transition Field $M^{\left(m\right)}\in R^{L\times L}$ is generated by mapping the transition probabilities back to the time domain:

(13) $$M^{\left(m\right)}\left(a,b\right)=P_{s_{a},s_{b}}^{\left(m\right)}$$

where $s_{a}$ and $s_{b}$ denote the quantile states corresponding to time indices *a* and *b*, respectively. In this way, each element of the MTF encodes the transition relationship between two time instants, enabling the image to capture both local temporal dependencies and global dynamic evolution patterns.

It should be noted that the MTF is fundamentally a numerical matrix rather than an image. However, by interpreting its values as pixel intensities, the matrix can be visualized and processed as a grayscale image. To meet the input requirement of Tri-CrossViT, each MTF image is resized to a fixed resolution of 224×224 using bilinear interpolation:

(14) $${\tilde{M}}^{\left(m\right)}=R_{\text{bilinear}}\left(M^{\left(m\right)}\right)$$

As described in subsection B, three IMFs have been selected by statistical measures. Thus, after MTF, three images can be obtained. The three resized images are then mapped into the R, G, and B channels of an RGB image, respectively. Specifically, the image from the IMF selected by kurtosis is mapped to the R channel; the image from the IMF selected by energy is mapped to the G channel; and the image from the IMF selected by the Pearson correlation coefficient is mapped to the B channel, producing a single RGB image.

To better illustrate CKEPM, the feature extraction process by using the CWRU bearing fault dataset is shown in Figure 1. The specific steps are as follows:

1. The raw vibration signal was first divided into non-overlapping training, validation, and test blocks with a ratio of 7:2:1 according to the chronological sample order, without random shuffling. The sliding-window segmentation was then performed independently within each subset using a window length of 1024 samples and 50% overlap (a stride of 512 samples). Importantly, no sliding window was allowed to cross the boundaries between the training, validation, and test subsets. Therefore, overlapping windows were generated only within the same subset, preventing shared temporal samples caused by the sliding-window operation from appearing in different subsets. Each resulting signal segment was subsequently normalized independently.

2. CEEMDAN was applied to each time-domain segment in Step 1 to obtain IMF components.

3. We calculated the kurtosis of the IMF components obtained from the decomposition of each segment in Step 2, and select the IMF component with the largest kurtosis for each segment.

4. Calculate the energy of the IMF components obtained from the decomposition of each segment in Step 2, and select the IMF component with the largest energy for each segment.

5. Compute the Pearson correlation coefficient between each IMF component obtained from the decomposition of each segment in Step 2 and the segment itself, and select the IMF component with the largest correlation coefficient for each segment.

6. Perform MTF transformation on the selected IMFs from Steps 3, 4, and 5, respectively, and resize each resulting image to 224×224 using bilinear interpolation.

7. Assign the image from Step 3 to the R channel, the image from Step 4 to the G channel, and the image from Step 5 to the B channel. This produces a 224×224 RGB image to be used as input for Tri-CrossViT.

**Figure 2:**
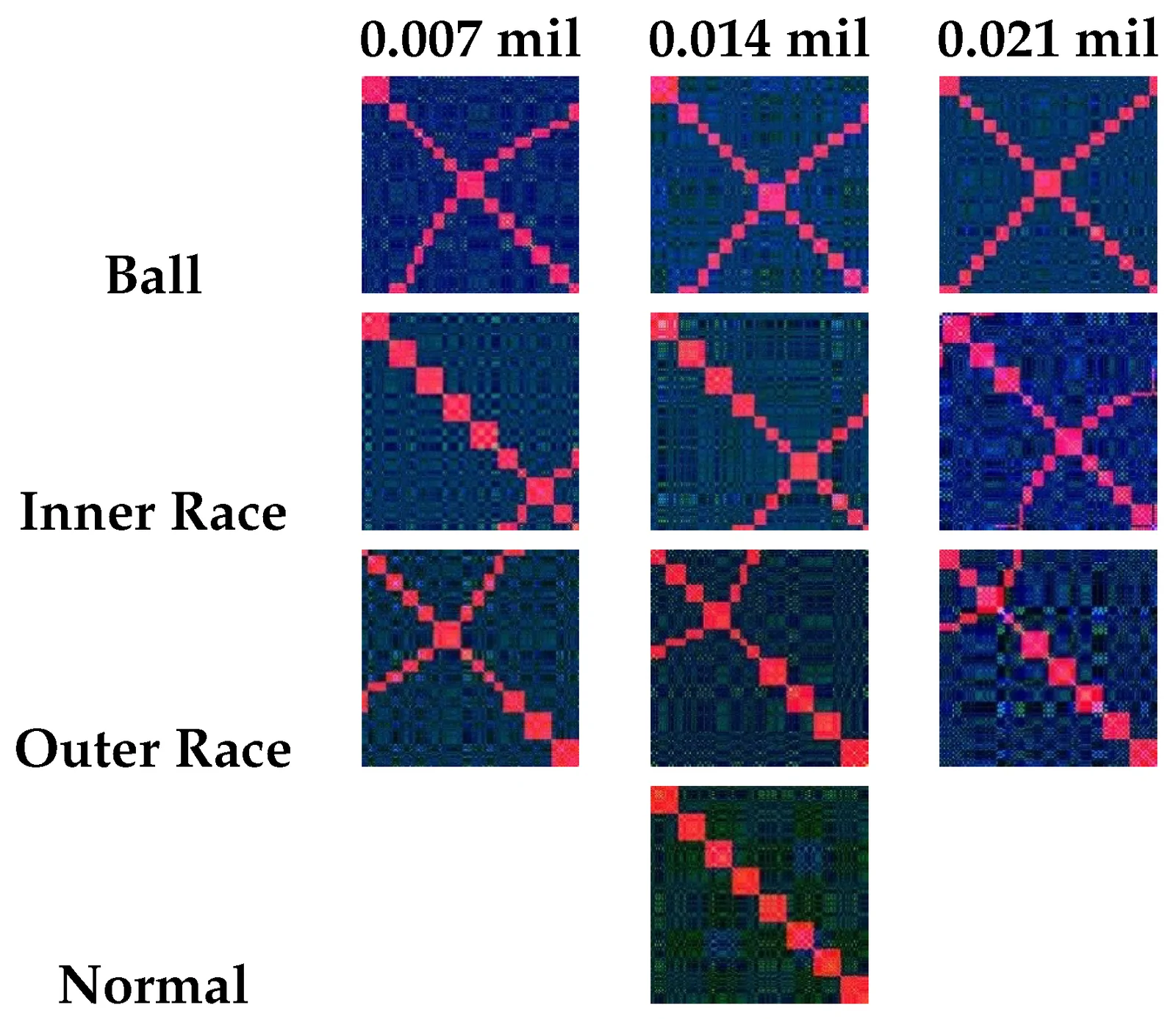
Representative CKEPM representations under different bearing conditions in the CWRU dataset. The columns correspond to fault diameters of 0.007, 0.014, and 0.021 mil, while the rows represent ball, inner-race, and outer-race faults. The normal condition is also included for comparison.

Examples of images corresponding to different fault conditions in the CWRU bearing fault dataset are presented in Figure 2. Here, “0.007 mil, 0.014 mil, 0.021 mil” denote fault diameters; “Ball, Inner Race, Outer Race” denote fault locations; and “Normal” denotes the healthy condition.

## 3. Tri-CrossViT

**Figure 3:**
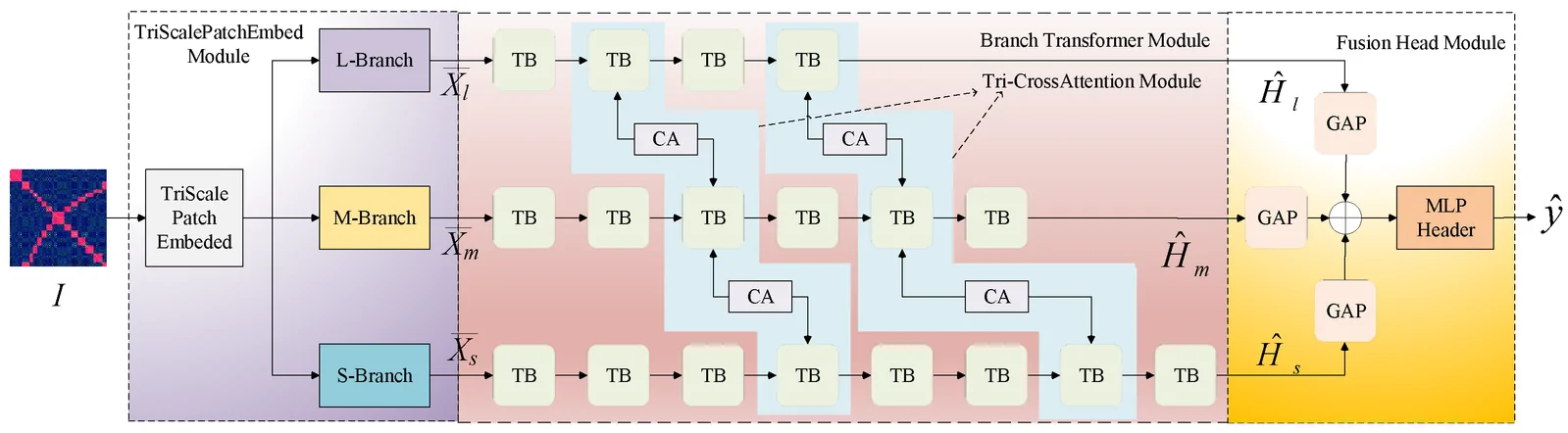
The overall architecture of the proposed Tri-CrossViT model, where *I* denotes the input CKEPM image; ${\bar{X}}_{i},i\in \left\{s,m,l\right\}$ are the multi-scale token sequences after patch embedding and positional encoding; the subscripts s, m, and l denote the small-, medium-, and large-scale branches; TB denotes the Transformer Block; CA denotes the cross-attention; ${\hat{H}}_{i},i\in \left\{s,m,l\right\}$ are the branch outputs after intra- and cross-branch attention; and $\hat{y}$ is the predicted fault-class score vector.

The proposed Tri-CrossViT network is the core component of the diagnostic pipeline. It takes the CKEPM RGB time–frequency image as input to predict the bearing fault category. As shown in Figure 3, the model consists of four modules: TriScalePatchEmbed, Branch Transformer, Tri-CrossAttention and Fusion Head.

### 3.1. TriScalePatchEmbed Module

The TriScalePatchEmbed module is designed to construct a triple-resolution tokenized representation of the input image. This design is motivated by the intrinsic multi-scale structure of bearing vibration in the time–frequency domain, where impulsive impacts, resonant modulations, and global frequency-energy distributions coexist but manifest at significantly different spatial granularities. A single-scale tokenizer would inevitably overemphasize the model toward either local details or global structure, whereas a triple-scale formulation has the capability to preserve relevant fault characteristics from the beginning of the processing pipeline.

Given the input image $I\in R^{3\times 224\times 224}$, three convolutional projections are applied as patchifying operators with different receptive fields. Specifically, The small-scale branch employs a 7×7 kernel with stride 7, capturing highly localized variations associated with abrupt fault-induced impulses or periodic micro-impacts; the medium-scale branch employs a 14×14 kernel with stride 14, which is well suited for representing the modulation effects typically caused by structural resonances; the large-scale branch employs a 28×28 kernel with stride 28, emphasizing the global distribution of spectral energy and the slow-varying envelope structures associated with bearing wear and progression.

The resulting feature maps of small-, medium-, and large-scale branches are

(15) $$\begin{array}{cc} F_{s} & \in R^{192\times 32\times 32}\\ F_{m} & \in R^{128\times 16\times 16}\\ F_{l} & \in R^{96\times 8\times 8} \end{array}$$

where the channel dimensions 192, 128, and 96 correspond to the embedding dimensions of the three branches.

The resulting feature maps are obtained by encoding the input image at three distinct spatial resolutions. Each spatial position in the feature map corresponds to a patch-level representation. To convert the feature maps into a sequence suitable for Transformer processing, the spatial dimensions are flattened and transposed, producing the following token sequences:

(16) $$\begin{array}{cc} X_{s} & \in R^{1024\times 192}\\ X_{m} & \in R^{256\times 128}\\ X_{l} & \in R^{64\times 96} \end{array}$$

where $X_{i}$ denotes the token sequence of branch *i*.

Besides, a learnable positional encoding ${PE}_{i}$ is added to each sequence. This yields three token sequences aligned in structure but distinct in resolution, ensuring that subsequent modules can access complementary fault-related features.

(17) $$\bar{X_{i}}=X_{i}+{PE}_{i},\;\;i\in s,m,l$$

where $\bar{X_{i}}$ denotes the final embedded token sequence of branch *i*, which serves as the input to the subsequent Branch Transformer module.

**Figure 4:**
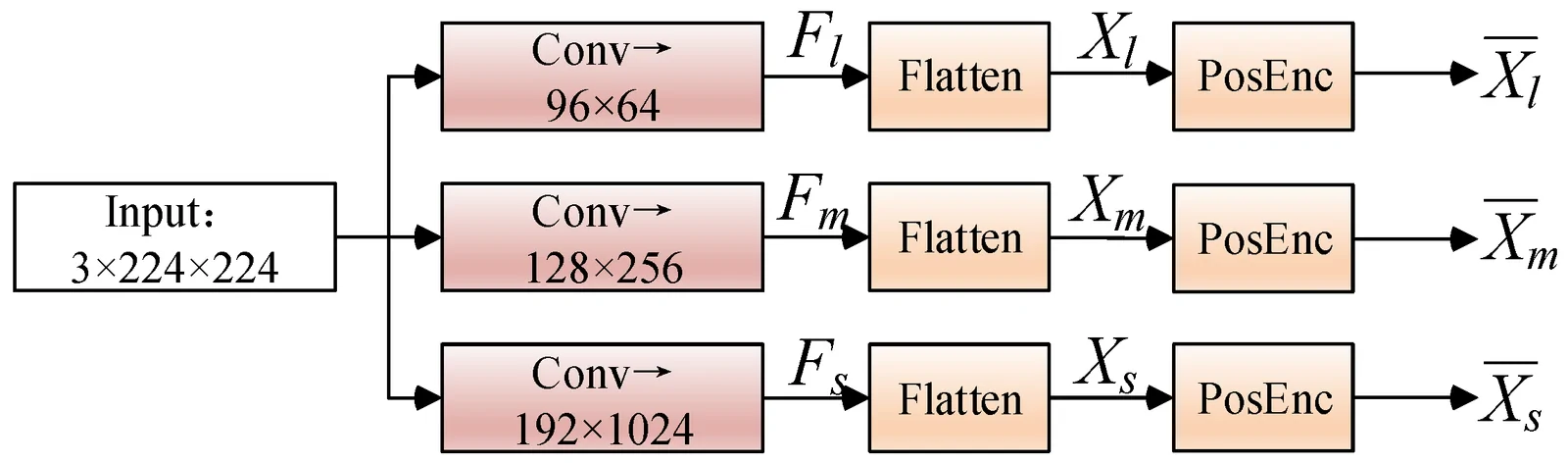
The structure of the TriScalePatchEmbed module, where the input image is encoded into three feature maps at different spatial resolutions.

The structure of the TriScalePatchEmbed module is illustrated in Figure 4.

### 3.2. Branch Transformer Module

The purpose of the Branch Transformer module is to independently model the intra-scale dependencies within each token sequence. This separation is essential because features extracted at different resolutions exhibit different structural characteristics: high-resolution tokens often encode abrupt, localized non-stationarities, whereas low-resolution tokens tend to carry smoother contextual information. Modeling them jointly would blur these scale-specific differences.

Therefore, each branch is equipped with a dedicated Transformer encoder with depth $L_{i}$, allowing each scale to form its own hierarchical representation before any cross-scale interaction occurs.

Given the initial tokens $Z_{i}^{\left(0\right)}=\bar{X_{i}}$, each Transformer block applies a pre-norm multi-head self-attention (MSA) followed by a feed-forward MLP, both with residual connections:

(18) $$\begin{array}{c} {\tilde{Z}}_{i}^{\left(\ell \right)}=Z_{i}^{\left(\ell \right)}+{\text{MSA}}_{i}\left(\text{LN}(Z_{i}^{\left(\ell \right)})\right)\\ Z_{i}^{\left(\ell +1\right)}={\tilde{Z}}_{i}^{\left(\ell \right)}+{\text{MLP}}_{i}\left(\text{LN}({\tilde{Z}}_{i}^{\left(\ell \right)})\right) \end{array}$$

where $\ell =0,1,\ldots ,L_{i}-1$, LN(·) denotes Layer Normalization, ${\text{MSA}}_{i}\left(\cdot \right)$ denotes the multi-head self-attention of branch *i*, and ${\text{MLP}}_{i}\left(\cdot \right)$ denotes the feed-forward network.

The deeper stack assigned to the small-scale branch ($L_{s}$ = 8) reflects the complexity and abundance of fine-grained structures inherent in vibration signals. The medium-scale branch ($L_{m}$ = 6) balances local detail and global contextual information, while the large-scale branch ($L_{l}$ = 4) prioritizes global context with a shallower configuration.

**Figure 5:**
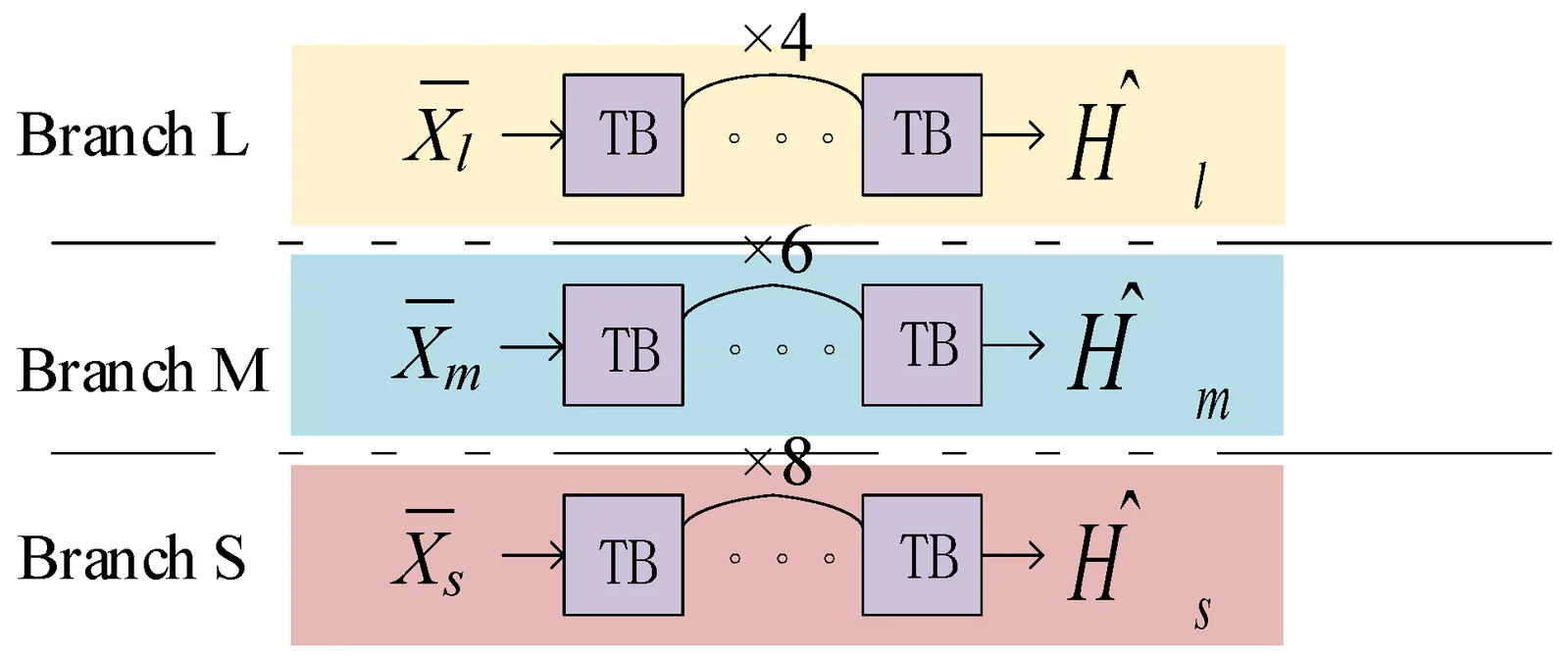
The architecture of the Branch Transformer module, where each branch performs intra-scale feature learning using multi-head self-attention and feed-forward networks.

Notably, Tri-CrossViT does not treat these encoders as fully independent branches; instead, cross-attention modules are interleaved into intermediate layers. This means $Z_{i}^{\left(\ell \right)}$ not only evolves through intra-scale self-attention but also receives cross-scale feature refinement, yielding the final enhanced ${\hat{H}}_{i}$. The architecture of the Branch Transformer module is shown in Figure 5.

### 3.3. Tri-CrossAttention Module

**Figure 6:**
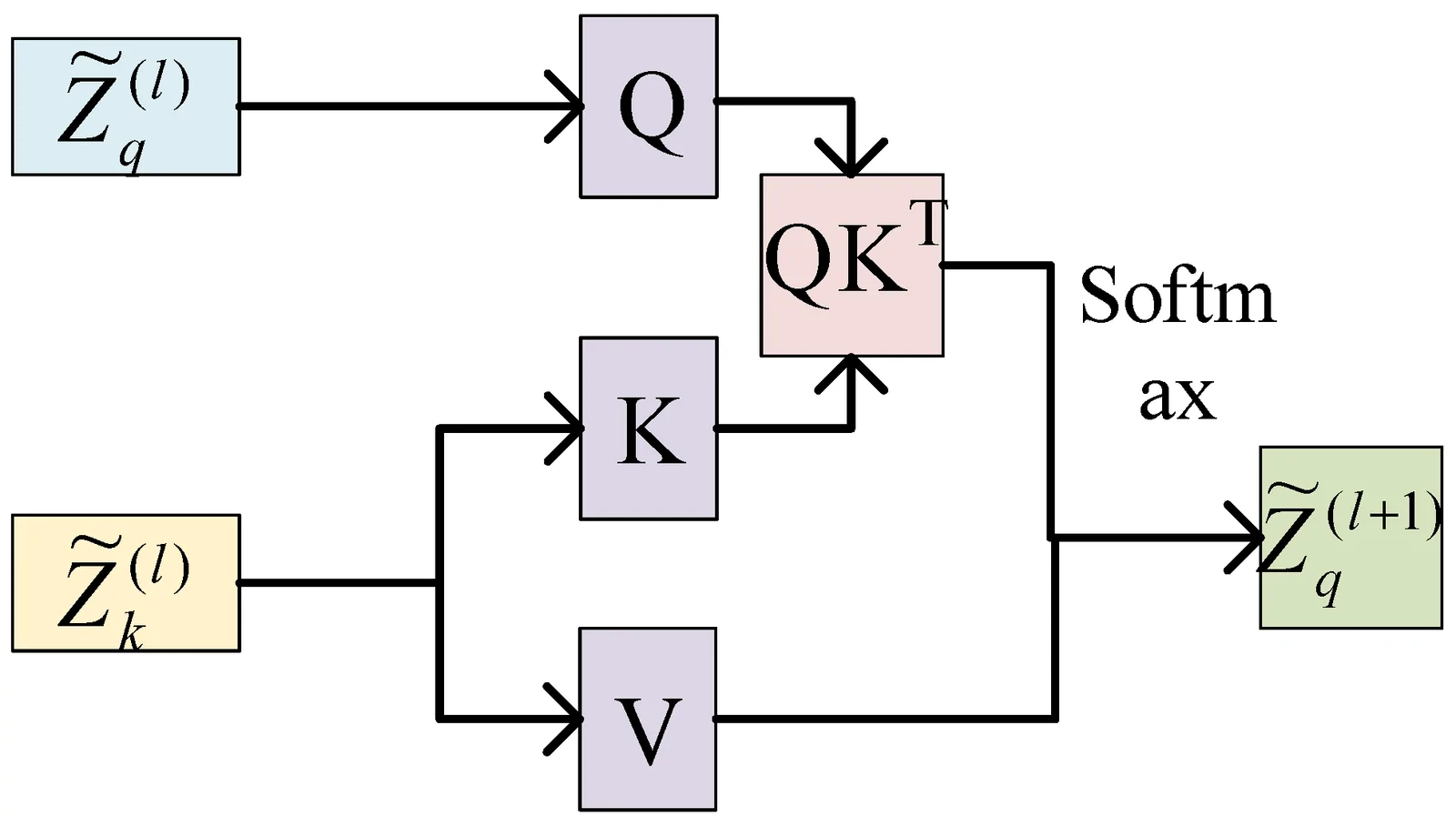
The cross-attention mechanism for inter-scale feature interaction, where information is exchanged between different branches to enhance representation consistency.

The Tri-CrossAttention mechanism is introduced to enable feature interaction across the three resolution branches constructed in Module B. While the self-attention operations within each branch capture intra-scale dependencies, many bearing fault characteristics, such as impulsive transients, modulation patterns, and harmonic structures are distributed across multiple time–frequency scales. To facilitate information exchange across branches, cross-attention operations are inserted into selected Transformer blocks of the three encoder stacks. The cross-attention interaction mechanism is illustrated in Figure 6.

Let $Z_{i}^{\left(\ell \right)},i\in \left\{s,m,l\right\}$, denote the token representation of branch *i* at the *ℓ*-th Transformer block. Following the self-attention operation described in Module B, the intermediate representation is updated as

(19) $${\tilde{Z}}_{i}^{\left(\ell \right)}=Z_{i}^{\left(\ell \right)}+{\text{MSA}}_{i}\left(\text{LN}(Z_{i}^{\left(\ell \right)})\right).$$

These intermediate tokens serve as the inputs to the cross-attention operation.

For a query sequence ${\tilde{Z}}_{q}^{\left(\ell \right)}$ and a key/value sequence ${\tilde{Z}}_{k}^{\left(\ell \right)}$, the cross-attention mechanism first projects the token representations into a shared interaction space:

(20) $$\begin{array}{c} Q={\tilde{Z}}_{q}^{\left(\ell \right)}W_{q}\\ K={\tilde{Z}}_{k}^{\left(\ell \right)}W_{k}\\ V={\tilde{Z}}_{k}^{\left(\ell \right)}W_{v} \end{array}$$

where $W_{q}\in R^{D_{q}\times d_{c}}$, and $W_{k}\in R^{D_{k}\times d_{c}}$, and $W_{v}\in R^{D_{k}\times d_{c}}$ are learnable projection matrices, and $d_{c}$ denotes the cross-attention embedding dimension.

The attention weights are computed using scaled dot-product attention:

(21) $$A=\text{softmax}(\frac{{QK}^{T}}{\sqrt{d_{c}}})$$

where $A\in R^{N_{q}\times N_{k}}$ describes the interaction between tokens of the two branches. The cross-attention output is then obtained as

(22) O=AV

The result is projected back to the query branch dimension and incorporated through a residual connection:

(23) $${\bar{Z}}_{q}^{\left(\ell \right)}={\tilde{Z}}_{q}^{\left(\ell \right)}+OW_{o}$$

where $W_{o}\in R^{d_{c}\times D_{q}}$ is the output projection matrix.

Finally, a feed-forward network is applied to produce the updated token representation of the query branch:

(24) $$Z_{q}^{\left(\ell +1\right)}={\bar{Z}}_{q}^{\left(\ell \right)}+{\text{MLP}}_{q}\left(\text{LN}({\bar{Z}}_{q}^{\left(\ell \right)})\right).$$

Thus, each cross-attention block updates the token representation of one branch by incorporating contextual information from another branch.

In Tri-CrossViT, cross-attention operations are inserted at multiple depths of the three Transformer encoder stacks to progressively integrate multi-scale information. The medium-scale branch plays a central role in the interaction process, providing a balanced representation between fine-scale details and global structures. Specifically, the medium branch receives complementary information from both the small and large branches:

(25) $$\begin{array}{c} m^{\left(3\right)}\leftarrow CA\left(m^{\left(3\right)},s^{\left(3\right)}\right)+CA\left(m^{\left(3\right)},l^{\left(2\right)}\right)\\ m^{\left(5\right)}\leftarrow CA\left(m^{\left(5\right)},s^{\left(4\right)}\right)+CA\left(m^{\left(5\right)},l^{\left(3\right)}\right) \end{array}$$

where CA(·,·) denotes the cross-attention operation, and the superscripts indicate the corresponding Transformer block indices. This design ensures that the medium branch captures fault signatures spanning multiple resolutions, which is important because mid-scale representations often encode modulation effects tightly coupled with both impulsive and global behaviors.

Simultaneously, both the small and large branches are updated using contextual information from the mid-scale branch:

(26) $$\begin{array}{c} s^{\left(4\right)}\leftarrow m^{\left(3\right)},s^{\left(7\right)}\leftarrow m^{\left(5\right)}\\ l^{\left(2\right)}\leftarrow m^{\left(3\right)},l^{\left(4\right)}\leftarrow m^{\left(5\right)}. \end{array}$$

Through these bidirectional interactions, the small branch benefits from contextual modulation information while the large branch gains improved alignment with localized transient structures.

After the Transformer encoder stacks complete all $L_{i}$ blocks with interleaved cross-attention operations, the final token representations of the three branches are obtained as

(27) $$\begin{array}{cc} {\hat{H}}_{s} & =Z_{s}^{\left(L_{s}\right)}\\ {\hat{H}}_{m} & =Z_{m}^{\left(L_{m}\right)}\\ {\hat{H}}_{l} & =Z_{l}^{\left(L_{l}\right)} \end{array}$$

These representations incorporate both intra-scale modeling (via self-attention) and inter-scale interaction (via cross-attention). The resulting feature sequences are subsequently sent to the Fusion Head for global feature aggregation and final fault classification.

### 3.4. Fusion Head Module

The Fusion Head module converts the triple-scale representations into a compact and discriminative feature vector suitable for classification. After the intra- and cross-scale processing in previous modules, the token sequences ${\hat{H}}_{i}$ contain rich feature representations distributed across scales. To convert these sequences into fixed-length vectors, global average pooling (GAP) is applied:

(28) $$f_{i}=\text{GAP}\left({\hat{H}}_{i}\right),\;\;i\in \left\{s,m,l\right\}.$$

This operation retains the dominant feature characteristics of each branch while eliminating token-wise redundancy.

Then, the pooled vectors are concatenated:

(29) $$f=\left[f_{s};f_{m};f_{l}\right]\in R^{D_{s}+D_{m}+D_{l}}$$

which effectively combines small-, mid-, and global-scale descriptions into a unified embedding. A LayerNorm is applied to normalize feature activations within each sample and reduce scale discrepancies across feature dimensions introduced by triple-branch feature fusion, followed by a two-layer MLP:

(30) $$\begin{array}{cc} h & =\text{GELU}\left(W_{1}\text{LN}\left(f\right)+b_{1}\right)\\ \hat{y} & =W_{2}h+b_{2} \end{array}$$

where $W_{1}$ and $W_{2}$ are the weight matrices of the classifier, and $b_{1}$ and $b_{2}$ are the corresponding bias terms.

**Figure 7:**
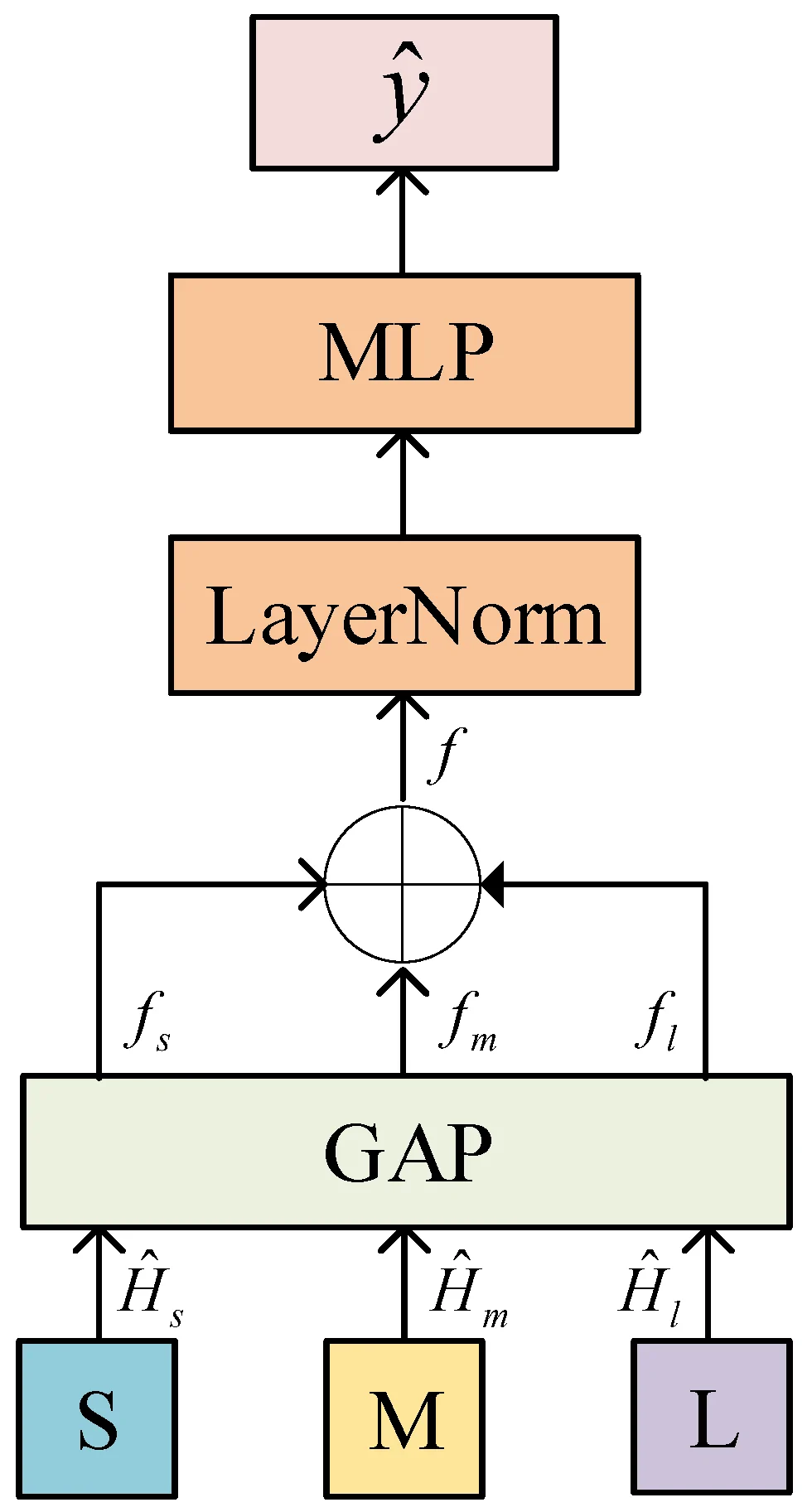
The Fusion Head module, where multi-scale features are aggregated via global average pooling and mapped to the final classification output.

Here, most representation learning and cross-scale integration are performed in previous module because this classifier remains lightweight while still providing sufficient discriminative capacity. The overall structure of the Fusion Head module is shown in Figure 7.

## 4. Experimental Analysis

The experiments are implemented in Python 3.9 using PyTorch 2.1.0+cu121. The experimental hardware is as follows: CPU: Intel i5-13400F; GPU: NVIDIA GeForce RTX 4060 Ti; RAM: 16 GB. The complete architectural configuration of Tri-CrossViT is summarized in the Table 1 and Table 2, respectively. The first table presents the basic structural parameters of the three-scale branches, while the second summarizes the cross-scale interaction settings.

### 4.1. Experimental Setup

Dataset 1: The experimental data come from dedicated tests at Case Western Reserve University and cover bearing vibration measurements under both normal operating conditions and multiple fault states. Bearing models include SKF6205 at the drive end and SKF6203 at the fan end, ensuring data diversity and practical relevance. This study primarily uses the drive-end acceleration data sampled at 48 kHz. Fault locations include inner race, outer race, and rolling element, with diameters of 0.007 mil, 0.014 mil, and 0.021 mil, resulting in 9 distinct fault types plus 1 normal condition, for a total of 10 classes. The details are given in the Table 3:

The CWRU dataset is partitioned into training, validation, and test sets containing 6300, 1800, and 900 samples, respectively.

Dataset 2: The experimental data come from the Paderborn University bearing dataset (PU). All tested bearings are model 6203. Faulty bearings include artificially induced damage and real damage, with damage severity levels from 1 to 5. Vibration signals are sampled at 64 kHz. In this study, we primarily used the bearings with artificially induced damage; the damage locations are the outer race and the inner race, with severity levels 1–2, resulting in four fault types plus one normal condition. Details are given in Table 4:

The PU dataset is partitioned into training, validation, and test sets containing 35,000, 10,000, and 5000 samples, respectively.

### 4.2. Model Performance Metrics

To comprehensively evaluate the classification performance of the model, multiple evaluation metrics are used, including precision (P), recall (Recall), F1 score (F1 score), validation-set classification accuracy, and the confusion matrix. The specific formulas are as follows:

(31) $$\begin{array}{cc} \text{precision} & =\frac{TP}{TP+FP}\\ \text{recall} & =\frac{TP}{TP+FN}\\ F1 & =2\times \frac{\text{precision}\times \text{recall}}{\text{precision}+\text{recall}}\\ AP & =\int_{0}^{1}p(r)dr. \end{array}$$

Here, TP, FP, FN, *p* and *r* denote true positives, false positives, false negatives, precision, and recall, respectively.

### 4.3. Analysis of Experimental Results

Experiment 1: Comparison of Different IMF Selection Criteria. To evaluate the contribution of each statistical criterion to IMF selection, seven selection strategies were compared under the same experimental conditions. Only the IMF selection criterion was changed, whereas CEEMDAN decomposition, MTF encoding, image resizing, dataset partitioning, the ResNet50 classifier, and the training settings were kept unchanged. For a single-criterion strategy, the MTF generated from the selected IMF was replicated across the R, G, and B channels. For a two-criterion strategy, the two corresponding MTFs were assigned to the first two channels, while their element-wise mean was assigned to the third channel. The results are summarized in Table 5.

As shown in Table 5, the joint Kurtosis–Energy–Pearson strategy achieves the highest validation accuracies, namely 96.86% on CWRU and 97.58% on PU. Among the single-criterion strategies, Kurtosis produces the best results, reaching 91.83% and 93.07%, respectively. This confirms that impulsiveness is an important indicator of bearing faults. However, Kurtosis alone remains 5.03% lower than the proposed strategy on CWRU and 4.51% lower on PU. Energy and Pearson correlation characterize different properties of an IMF. Neither criterion alone is sufficient to comprehensively preserve fault-related information. The two-criterion strategies generally improve over their corresponding single-criterion alternatives, but they still perform below the three-criterion strategy. In particular, the best two-criterion result is obtained by K+P, with 93.77% on CWRU, and by K+E, with 94.80% on PU. Nevertheless, the proposed K+E+P strategy improves these results by 3.09% and 2.78%, respectively. These results demonstrate that the joint use enables more comprehensive identification of fault-sensitive IMFs and produces more discriminative CKEPM representations than either single-criterion or two-criterion selection.

Experiment 2: Comparison of Feature Extraction Methods. To evaluate the feature extraction capability of CKEPM, three commonly used methods—GAF, SDP, Recurrence graph, and the proposed CKEPM—are compared under the same ResNet backbone and identical training hyperparameters. The results are shown in Table 6.

From Table 6, it can be seen that the average classification accuracy of CKEPM is 96.86%, outperforming GAF (95.71%), Recurrence Graph (93.75%) and SDP (91.15%). This result indicates that the CKEPM method, which selects and fuses IMFs based on kurtosis combined with energy and correlation, can more effectively preserve impulsive and band-specific fault features in bearing signals, thereby improving the discriminative performance of convolutional networks.

Experiment 3: Comparison of Classification Models. Comparative experiments are conducted on the CWRU and PU bearing fault datasets using several representative deep learning models, including MobileNetV3, CrossViT, Swin Transformer, MobileViT, Transformer-BiLSTM, and the proposed Tri-CrossViT. These models cover representative lightweight CNN, multi-scale Transformer, hierarchical Transformer, and hybrid Transformer-based architectures, providing a comprehensive comparison with the proposed method. All experiments are conducted under the same data preprocessing, training, and evaluation protocols. The average test accuracy of each model over 10 repeated runs on the CWRU and PU bearing fault datasets is presented in Table 7. To ensure a controlled comparison, all competing models are trained under the same data preprocessing, data partitioning, and training hyperparameter settings. This unified configuration is intentionally adopted to reduce the influence of model-specific hyperparameter tuning and to facilitate a direct comparison of the model differences among the competing models. The detailed hyperparameter configurations are summarized in Table 8.

It can be observed that the test accuracies of CrossViT on the CWRU and PU bearing fault datasets are 94.73% and 97.45%, respectively; MobileNetV3 achieves 90.92% and 94.27%; Swin Transformer achieves 94.58% and 98.19%; MobileViT achieves 97.36% and 98.44%; Transformer-BiLSTM achieves 96.16% and 97.73%; and the proposed Tri-CrossViT achieves 98.87% and 99.51%, obtaining the highest test accuracy on both datasets.

Specifically, compared with CrossViT, Tri-CrossViT improves the test accuracy by 4.14% on the CWRU dataset and 2.06% on the PU dataset. Compared with MobileNetV3, the improvements reach 7.95% and 5.24% on the CWRU and PU datasets, respectively. Compared with Swin Transformer, Tri-CrossViT achieves improvements of 4.29% and 1.32%, while the corresponding improvements over MobileViT are 1.51% and 1.07%. Compared with Transformer-BiLSTM, Tri-CrossViT further improves the test accuracy by 2.71% on CWRU and 1.78% on PU. Although Transformer-BiLSTM combines the long-range dependency modeling capability of Transformer with the bidirectional temporal feature extraction capability of BiLSTM, its performance remains lower than that of Tri-CrossViT on both datasets. These results demonstrate that the triple-scale design and intermediate cross-scale interactions adopted in Tri-CrossViT provide effective and stable improvements for bearing fault diagnosis. Notably, the comparison between CrossViT and Tri-CrossViT further demonstrates the benefit of introducing a third branch to complement the intermediate-scale representation and performing multiple intermediate-layer cross-scale interactions to enhance feature discrimination.

From an architectural perspective, the comparative results also reflect differences in the feature-learning strategies of these models. Lightweight models such as MobileNetV3 and Transformer-BiLSTM require relatively low computational cost, but achieve lower test accuracy on both datasets. CrossViT employs a two-branch multi-scale architecture, whereas Tri-CrossViT introduces an additional intermediate-scale branch together with multiple cross-scale interactions to enhance complementary feature exchange. Although Swin Transformer achieves competitive performance, it requires substantially more parameters and computational cost. These comparisons further indicate that the observed performance differences are associated not only with model capacity, but also with the strategies used for multi-scale feature representation and information interaction.

The core reasons for the performance improvements of Tri-CrossViT are mainly threefold: First, TriScalePatchEmbed preserves small-, medium-, and large-grained features in parallel at the input, enabling us to retain complementary multi-scale information from the very first layer, which may be difficult for single-scale or two-branch methods to achieve simultaneously. Second, Tri-CrossAttention inserts bidirectional cross-scale attention across multiple intermediate layers, allowing information at different scales to be supplemented and corrected at multiple levels, which enhances discrimination of weak fault signals and noise artifacts. Third, by allocating different depths and embedding capacities to different branches, the model achieves a rational capacity allocation, yielding efficient representational power.

Table 7 further compares the model complexity and computational cost of different methods. Compared with lightweight models such as MobileNetV3, the proposed Tri-CrossViT introduces additional computational overhead due to its triple-scale architecture and cross-attention mechanism. Transformer-BiLSTM also exhibits relatively low computational complexity, requiring approximately 1.16 million parameters and 0.24 GFLOPs, but its test accuracies are 2.71% and 1.78% lower than those of Tri-CrossViT on the CWRU and PU datasets, respectively. This indicates that lower computational complexity does not necessarily lead to better diagnostic performance. Although Tri-CrossViT requires more computation than lightweight models, it uses substantially fewer parameters and lower computational cost than Swin Transformer while achieving the highest test accuracy on both datasets. These results demonstrate that Tri-CrossViT provides a favorable trade-off between diagnostic performance and model complexity.

Tri-CrossViT achieved favorable results on both the CWRU and PU bearing fault datasets. The performance of Tri-CrossViT for different fault types on the two datasets is presented in Table 9 and Table 10, including precision, recall, and F1 score.

From Table 9, Tri-CrossViT exhibits excellent performance on the CWRU dataset. Notably, for the Normal class the model achieves 100% precision, recall, and F1 score, indicating perfect discrimination of healthy operation. Specifically, at a defect diameter of 0.007 mils, the model shows stable recognition for rolling-element and inner-race faults: precision is 98.86% and 96.74%, recall is 96.67% and 98.89%, and F1 score is 97.75% and 97.80%, respectively. This demonstrates the ability of the model to effectively distinguish between different fault types. At 0.014 mils, the inner-race fault classification is outstanding, achieving 100% precision, recall, and F1 score, i.e., perfect classification for this category. At 0.021 mils, for inner-race and outer-race faults the model attains very high precision (100% and 98.90%) and recall (98.89% and 100%), with F1 scores close to perfect—indicating the model reliably captures and classifies the features of these fault types.

From Table 10, it can be seen that on the PU bearing dataset Tri-CrossViT also demonstrates excellent performance. The Normal class again reaches 100% precision, recall, and F1 score, confirming accurate normal-state identification. Performance across the other classes is also strong: precision and recall for all classes are approximately 98%, indicating that the model’s fault-classification capability on the PU dataset is very high.

**Figure 8:**
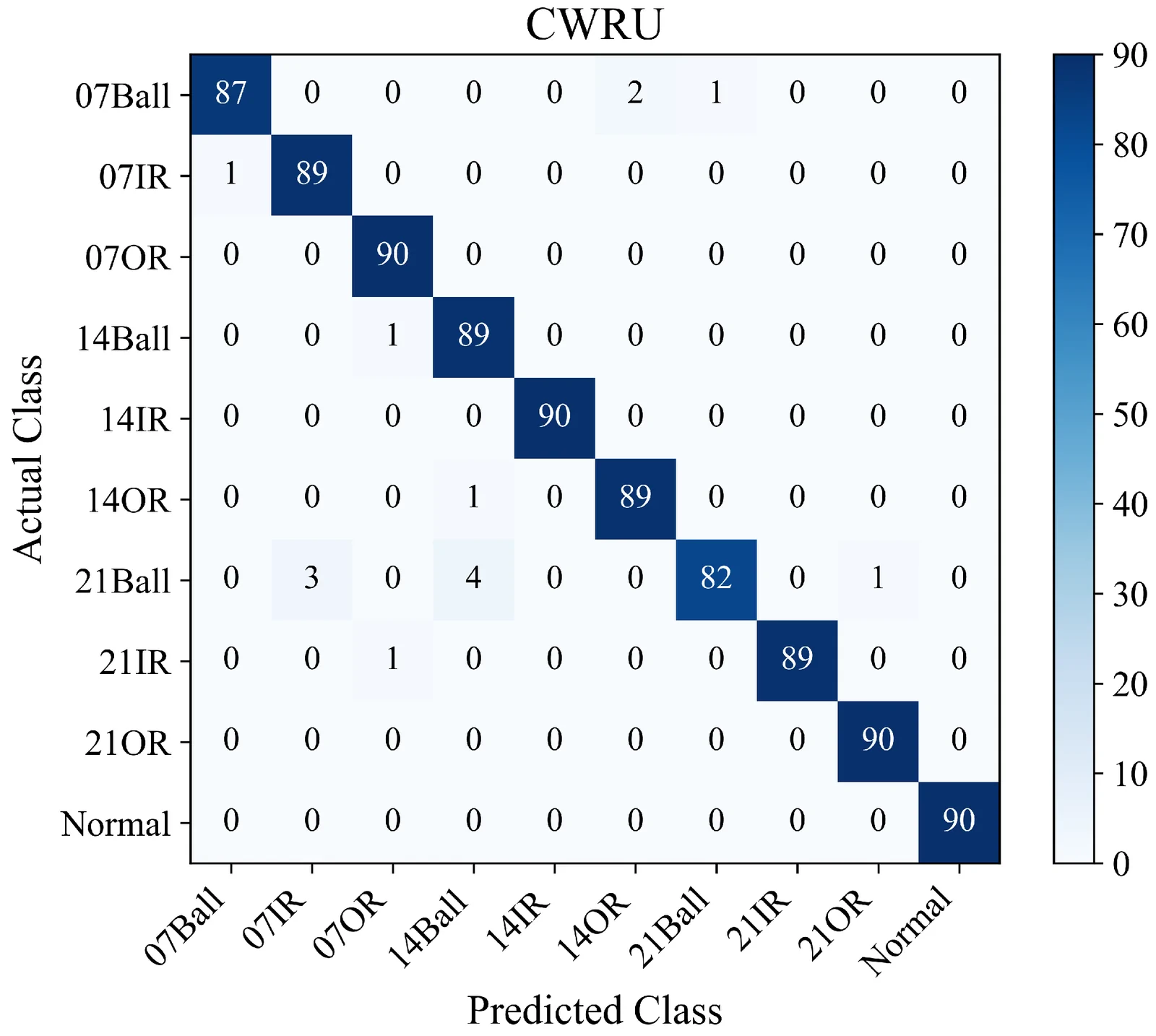
The confusion matrix obtained on the CWRU dataset, showing the classification performance of different fault categories.

**Figure 9:**
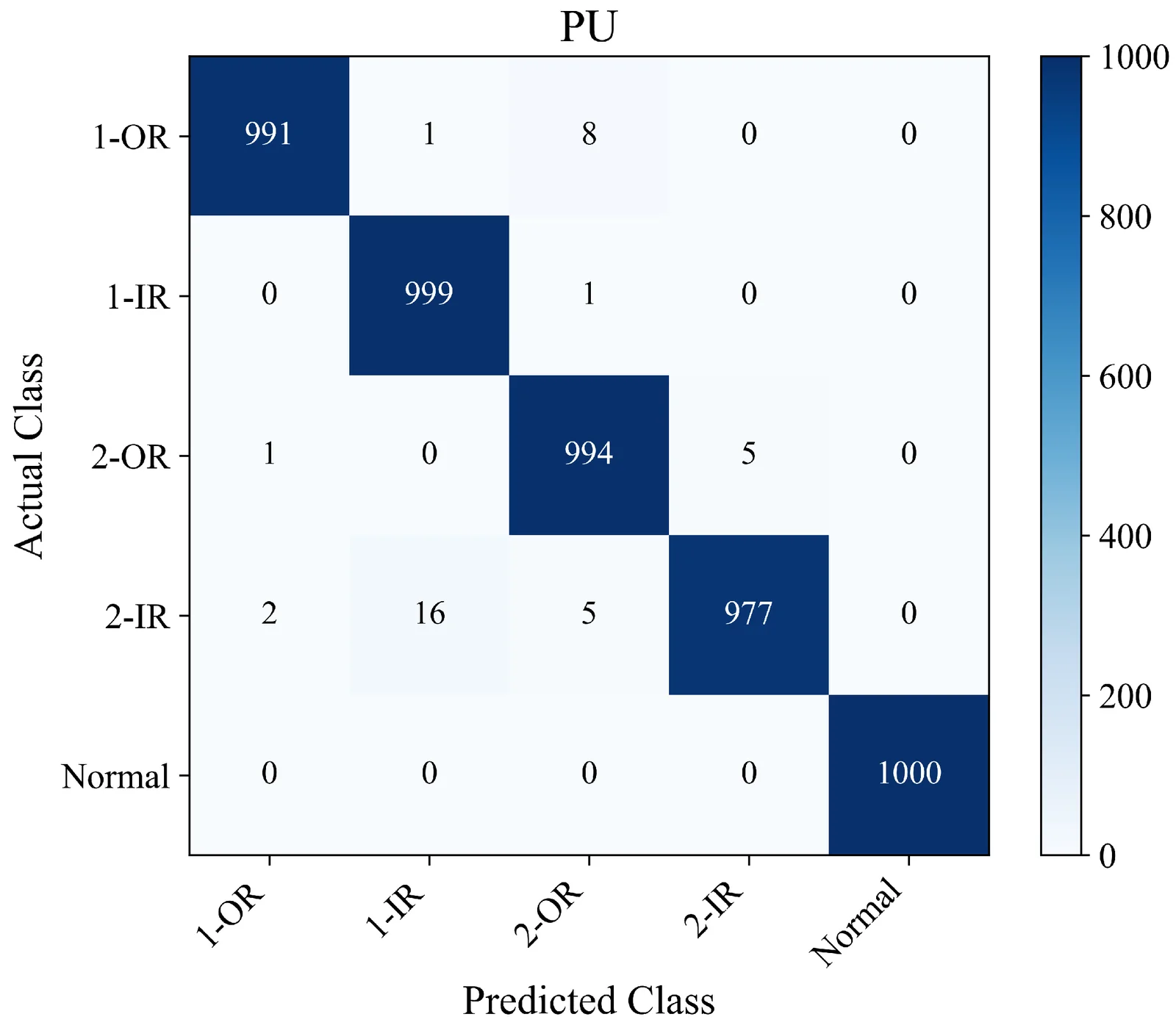
The confusion matrix obtained on the PU dataset, demonstrating the effectiveness of the proposed method under different fault conditions.

Additionally, the confusion matrix is one of the standard metrics for evaluating accuracy. The confusion matrix for the CWRU bearing fault dataset is of size 10×10, while that for the PU bearing fault dataset is 5×5. Confusion matrices provide a more intuitive way to assess model performance. The confusion matrices for the CWRU and PU datasets are shown in Figure 8 and Figure 9, respectively.

As shown in Figure 8 and Figure 9, the confusion matrices are predominantly diagonal, confirming the strong discriminative capability of the proposed method. A few misclassifications remain on the CWRU dataset, mainly between fault categories exhibiting similar vibration characteristics or comparable damage severities, whose encoded time–frequency representations share highly correlated feature patterns. In contrast, the confusion matrix on the PU dataset is nearly perfectly diagonal, indicating that the proposed framework can effectively distinguish different fault categories. Overall, the very limited number of classification errors demonstrates the robustness and effectiveness of the proposed CKEPM-Tri-CrossViT framework.

### 4.4. Ablation Study

To evaluate the contribution of the cross-scale interaction mechanism, three variants were compared under the same dataset partition and training settings: a model without cross-scale interaction, a model using only class-token interaction, and the proposed model using full-token cross-scale interaction. The results are summarized in Table 11.

The model without cross-scale interaction achieves only 80.58% accuracy, indicating that independent branch learning is insufficient for effectively integrating fault information at different scales. Introducing class-token interaction improves the accuracy to 88.81%, demonstrating the benefit of cross-branch information exchange. However, interaction through only the class tokens cannot fully exploit the complementary information distributed among the patch tokens. The proposed full-token interaction further increases the accuracy to 98.87%, confirming that dense cross-scale feature exchange is the main contributor to the performance improvement of Tri-CrossViT. Although the full-token strategy introduces additional computational cost, it provides a substantial gain in diagnostic accuracy.

## 5. Conclusions

To address the difficulty of extracting discriminative fault features from complex vibration signals and reliably identifying bearing fault types, this study proposed a pipeline from time-domain vibration signals to model discriminative outputs, including fault-feature extraction and fault-type identification. The pipeline mainly comprised a feature extraction method, termed CKEPM, which integrated CEEMDAN, statistical IMF selection, and Markov Transition Field encoding, together with a classification model based on Tri-CrossViT. First, CKEPM decomposed the raw signal into a set of IMFs using CEEMDAN and selected appropriate IMFs according to kurtosis, energy, and Pearson correlation criteria. Then, the selected IMFs were converted into matrices via MTF to form RGB images. Next, the resulting RGB images were fed into the proposed Tri-CrossViT, which employed small-, medium-, and large-scale parallel patch embeddings and incorporated a cross-scale attention mechanism at intermediate layers to achieve multi-level inter-branch information complementarity. Finally, a fusion head produced the predicted fault class.

Experimental results showed that this pipeline demonstrated excellent classification performance on both CWRU and PU bearing fault datasets, indicating that the proposed CKEPM feature-extraction method and the Tri-CrossViT diagnostic model had strong practical potential for fault diagnosis. Despite the promising results, several limitations remain. First, the proposed method was only validated on two public bearing datasets collected under relatively controlled laboratory conditions. Its robustness under variable operating conditions, severe environmental noise, and cross-domain scenarios requires further investigation. Second, although the proposed Tri-CrossViT achieved superior diagnostic performance, its multi-branch Transformer architecture inevitably introduced higher computational complexity than lightweight convolutional models, which may limit deployment on resource-constrained edge devices. Future work will therefore focus on lightweight network design, domain adaptation, and validation using real industrial datasets to further improve the practicality and generalization capability of the proposed framework.

## Figures and Tables

**Table 1:** Basic structural parameters of the three-scale branches.

Module	Parameter	Small Scale	Medium Scale	Large Scale
Patch Embedding	Kernel/Stride	7×7/7	14×14/14	28×28/28
Token Number	H×W	32×32	16×16	8×8
Embedding Dimension	Dim	192	128	96
Transformer Blocks	Depth	8	6	4
Multi-head Attention	Heads	4	4	4

**Table 2:** Parameters of the cross-scale attention module and classification head.

Module	Parameter Setting
Cross-scale attention dimension	256
Small-scale interaction positions	Layer 4 and Layer 7
Medium-scale interaction positions	Layer 3 and Layer 5
Large-scale interaction positions	Layer 2 and Layer 4

**Table 3:** CWRU fault categories.

Fault Diameter (mils)	Fault Type
	Inner Race
0.007	Ball
	Outer Race
	Inner Race
0.014	Ball
	Outer Race
	Inner Race
0.021	Ball
	Outer Race
–	Normal

**Table 4:** PU fault category.

Extent Damage (Level)	Fault Type
	OR
1	IR
	OR
2	IR
–	Normal

where “1” and “2” denote the artificial damage severity levels in the PU dataset, while “OR” and “IR” represent outer-race and inner-race faults, respectively.

**Table 5:** Validation accuracy under different IMF selection criteria.

IMF Selection Criterion	CWRU	PU
Kurtosis (K)	91.83%	93.07%
Energy (E)	88.13%	90.82%
Pearson Correlation (P)	86.93%	89.11%
Kurtosis + Energy	91.92%	94.80%
Kurtosis + Pearson Correlation	93.77%	94.59%
Energy + Pearson Correlation	91.32%	92.27%
Kurtosis + Energy + Pearson Correlation	96.86%	97.58%

**Table 6:** Comparison experiment of feature extraction methods.

Methods	GAF	SDP	Recurrence Graph	CKEPM
Accuracy	95.71%	91.15%	93.75%	**96.86%**

**Table 7:** Comparison of test accuracy, model complexity and computational cost.

Methods	Average Test Accuracy		Parameters	GFLOPs
	CWRU	PU		
CrossViT	94.73%	97.45%	6.72	1.29
MobileNetV3	90.92%	94.27%	1.52	0.06
Swin Transformer	94.58%	98.19%	86.75	15.14
MobileViT	97.36%	98.44%	6.96	1.44
Transformer-BiLSTM	96.16%	97.73%	1.16	0.24
Tri-CrossViT	98.87%	99.51%	10.62	10.08

**Table 8:** Unified training hyperparameter settings used for all comparison models.

Hyperparameter	Value
Optimizer	AdamW
Initial learning rate	$5\times 10^{-4}$
Batch size	32
Training epochs	300
Learning rate scheduler	Cosine Annealing
Weight decay	0.05
Image size	224×224
Input channels	3
Loss function	Cross-Entropy Loss
Label smoothing	0.1
Mixup	0.8
CutMix	1.0

**Table 9:** Performance metrics on CWRU.

Classes	Precision	Recall	F1 Score
07Ball	98.86%	96.67%	97.75%
07IR	96.74%	98.89%	97.80%
07OR	97.83%	100%	98.90%
14Ball	94.68%	98.89%	96.74%
14IR	100%	100%	100%
14OR	97.80%	98.89%	98.34%
21Ball	98.80%	91.11%	94.80%
21IR	100%	98.89%	99.44%
21OR	98.90%	100%	99.45%
Normal	100%	100%	100%

**Table 10:** Performance indicators on PU.

Classes	Precision	Recall	F1 Score
1-OR	99.70%	99.10%	99.40%
1-IR	98.33%	99.90%	99.11%
2-OR	98.61%	99.40%	99.00%
2-IR	99.49%	97.70%	98.59%
Normal	100%	100%	100%

**Table 11:** Ablation results of different cross-scale interaction strategies on the CWRU dataset.

Model Variant	Accuracy	GFLOPs	Parameters
Without cross-scale interaction	80.58%	7.36	6,125,288
CLS-token interaction	88.81%	8.07	10,620,200
Full-token interaction (Ours)	98.87%	10.08	10,619,386

## Data Availability

All raw experimental signals analyzed in this work come from two publicly available bearing datasets: the CWRU dataset https://engineering.case.edu/bearingdatacenter (accessed on 20 August 2026) and the PU dataset https://groups.uni-paderborn.de/kat/BearingDataCenter (accessed on 20 August 2026).

## References

[1] Li X., Ma Z., Yuan Z., Mu T., Du G., Liang Y., Liu J. (2024). A review on convolutional neural network in rolling bearing fault diagnosis. Meas. Sci. Technol..

[2] Chen X., Yang R., Xue Y., Huang M., Ferrero R., Wang Z. (2023). Deep transfer learning for bearing fault diagnosis: A systematic review since 2016. IEEE Trans. Instrum. Meas..

[3] Lei Y., Yang B., Jiang X., Jia F., Li N., Nandi A.K. (2020). Applications of machine learning to machine fault diagnosis: A review and roadmap. Mech. Syst. Signal Process..

[4] Hakim M., Omran A.A.B., Ahmed A.N., Al-Waily M., Abdellatif A. (2023). A systematic review of rolling bearing fault diagnoses based on deep learning and transfer learning: Taxonomy, overview, application, open challenges, weaknesses and recommendations. Ain Shams Eng. J..

[5] Khan A., Al Farid F., Junaid A., Siddique M.F., Iqbal A., Siddique M.S., Uddin J., Karim H.A., Husnain G. (2026). Early-warning industrial fault detection based on physics-guided residual learning and calibrated CRNNs. Sci. Rep..

[6] Siddique M.F., Ahmad Z., Kim J.M. (2023). Pipeline leak diagnosis based on leak-augmented scalograms and deep learning. Eng. Appl. Comput. Fluid Mech..

[7] Zhang Y., Xing K., Bai R., Sun D., Meng Z. (2020). An enhanced convolutional neural network for bearing fault diagnosis based on time–frequency image. Measurement.

[8] Chen Z., Mauricio A., Li W., Gryllias K. (2020). A deep learning method for bearing fault diagnosis based on cyclic spectral coherence and convolutional neural networks. Mech. Syst. Signal Process..

[9] Yuan L., Lian D., Kang X., Chen Y., Zhai K. (2020). Rolling bearing fault diagnosis based on convolutional neural network and support vector machine. IEEE Access.

[10] Shi L., Liu W., You D., Yang S. (2024). Rolling bearing fault diagnosis based on CEEMDAN and CNN-SVM. Appl. Sci..

[11] Tong A., Zhang J., Wang D., Xie L. (2024). Intelligent fault diagnosis of rolling bearings based on Markov transition field and mixed attention residual network. Appl. Sci..

[12] Ding S., Rui Z., Lei C., Zhuo J., Shi J., Lv X. (2023). A rolling bearing fault diagnosis method based on Markov transition field and multi-scale Runge-Kutta residual network. Meas. Sci. Technol..

[13] Ding Y., Jia M., Miao Q., Cao Y. (2022). A novel time–frequency Transformer based on self–attention mechanism and its application in fault diagnosis of rolling bearings. Mech. Syst. Signal Process..

[14] Dai X., Yi K., Wang F., Cai C., Tang W. (2024). Bearing fault diagnosis based on POA-VMD with GADF-Swin Transformer transfer learning network. Measurement.

[15] Wang Z., Xu Z., Cai C., Wang X., Xu J., Shi K., Zhong X., Liao Z., Li Q. (2024). Rolling bearing fault diagnosis method using time–frequency information integration and multi-scale TransFusion network. Knowl.-Based Syst..

[16] Ji M., Zhao G. (2024). DEViT: Deformable convolution-based Vision Transformer for bearing fault diagnosis. IEEE Trans. Instrum. Meas..

[17] Chen C.F.R., Fan Q., Panda R. (2021). CrossViT: Cross-attention multi-scale vision transformer for image classification. Proceedings of the IEEE/CVF International Conference on Computer Vision.

[18] Liu Z., Lin Y., Cao Y. (2021). Swin Transformer: Hierarchical vision transformer using shifted windows. Proceedings of the IEEE/CVF International Conference on Computer Vision.

[19] Wang W., Xie E., Li X. (2021). Pyramid vision transformer: A versatile backbone for dense prediction without convolutions. Proceedings of the IEEE/CVF International Conference on Computer Vision.

[20] Torres M.E., Colominas M.A., Schlotthauer G. (2021). A complete ensemble empirical mode decomposition with adaptive noise. Proceedings of the 2011 IEEE International Conference on Acoustics, Speech and Signal Processing (ICASSP).

[21] Wang Z., Oates T. (2015). Imaging time-series to improve classification and imputation. Proceedings of the Twenty-Fourth International Joint Conference on Artificial Intelligence (IJCAI).

